# A scoping review of studies applying the Nuffield’s ‘intervention ladder’ framework to assess the acceptability of diet and physical activity interventions

**DOI:** 10.1093/pubmed/fdaf156

**Published:** 2025-12-11

**Authors:** Sahana Ramamoorthy, Nazeem Muhajarine, Lise Gauvin

**Affiliations:** Department of Community Health and Epidemiology, College of Medicine, University of Saskatchewan, 104 Clinic Place, Saskatoon, Saskatchewan, S7N 2Z4, Canada; Saskatchewan Population Health and Evaluation Research Unit (SPHERU), University of Saskatchewan, 104 Clinic Place, Saskatoon, Saskatchewan, S7N 2Z4, Canada; Department of Community Health and Epidemiology, College of Medicine, University of Saskatchewan, 104 Clinic Place, Saskatoon, Saskatchewan, S7N 2Z4, Canada; Saskatchewan Population Health and Evaluation Research Unit (SPHERU), University of Saskatchewan, 104 Clinic Place, Saskatoon, Saskatchewan, S7N 2Z4, Canada; Department of Social and Preventive Medicine, School of Public Health, Université de Montréal, 7101 Park Ave, Montreal, Quebec H3N 1X9, Canada; Centre de Recherche du CHUM, Montréal, QC, Canada

**Keywords:** autonomy, health interventions, intervention ladder, intrusiveness, public acceptability

## Abstract

**Background:**

The Nuffield’s Intervention Ladder (NIL) framework casts public acceptability of health interventions based on their level of intrusiveness— how much they restrict personal autonomy and freedom of choice. This scoping review explores the application of the NIL framework in assessing public acceptability of diet and physical activity interventions, identifying key trends, gaps, and alignment with the framework’s conceptual underpinnings.

**Methods:**

We searched six databases (PubMed, Scopus, Medline, Embase, Science Direct, and Web of Science) and included 15 eligible studies. Data were charted and synthesized thematically and narratively.

**Results:**

The NIL framework was applied across different study designs, primarily post hoc, to categorize interventions based on their intrusiveness. Consistent with the framework, less intrusive interventions (information provision, enabling choice) were widely accepted. Moderately intrusive interventions (changing defaults, incentives, and disincentives) received mixed public acceptance, whereas highly intrusive interventions (restrict and eliminate choice) generally garnered lower public acceptability. Highly intrusive interventions were publicly acceptable when they are directed at children, or at industries. Across all intervention types, demographic and behavioural factors significantly influenced public acceptance.

**Conclusion:**

The NIL framework offers useful insights into how intrusiveness affects public acceptability of interventions. However, the review highlights that various factors influence acceptability in ways that extend the framework’s initial propositions.

## Background

Public health initiatives continue to focus on interventions that promote healthy diets and physical activity, striving to empower individuals to make healthier choices and foster environments that support them.^1–4^ At the heart of these interventions lie two critical pillars: evidence of effectiveness and ethical considerations.^5^ While there is a wealth of knowledge supporting the effectiveness of such interventions,^1^^,^^3^^,^^4^^,^^6–12^ there remains a notable gap in guidance on evaluating their ethical implications, particularly regarding public acceptability.^13^ One of the widely recognized tools for evaluating the public acceptability of public health interventions is the Nuffield’s Intervention Ladder (NIL) framework, developed by the Nuffield Council on Bioethics. Established in 1991, this UK-based body offers expert advice on ethical issues in science and medicine.^14^ Introduced in their 2007 report *Public Health: Ethical Issues*, the NIL framework provides a systematic approach for evaluating the justification and public acceptability of health interventions. Central to the framework is individual autonomy—the right of individuals to make decisions about their own lives. The NIL classifies interventions based on how they impact or restrict this fundamental autonomy.^15^

**Figure 1:**
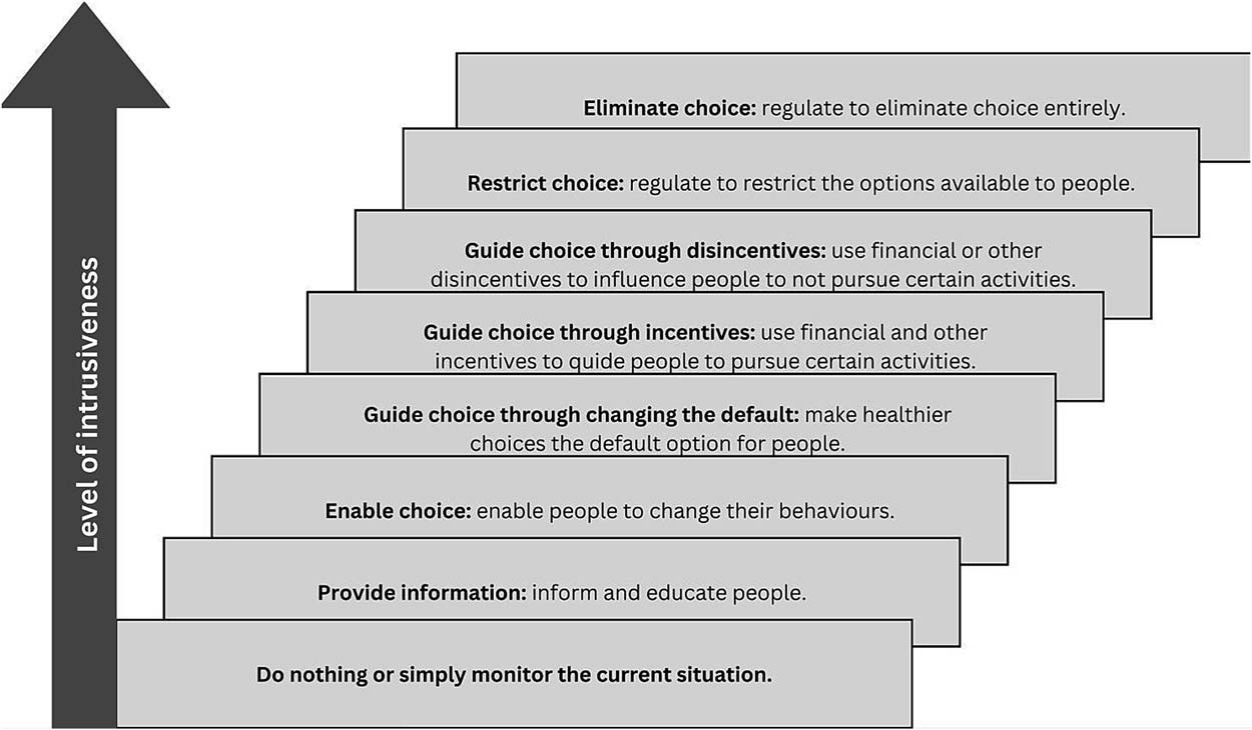
Nuffield’s ‘intervention ladder’ (NIL) (adapted from [14]).

The NIL framework classifies interventions into eight levels based on their degree of intrusiveness, from the least to the most intrusive (Fig. 1). Interventions at lower rungs of the ladder involve minimal intrusion on individual autonomy and demand greater personal agency. In contrast, interventions at the higher levels are more intrusive, often involving regulatory policies that limit personal freedoms and require minimum to no personal agency.^16^ The frameworks emphasize two key principles.^15^ First, interventions at higher levels must be strongly justified, with benefits clearly outweighing the potential loss of individual autonomy. Second, there is an inverse relationship between the intrusiveness of an intervention and its public acceptability: the more intrusive the intervention, the less likely it is to be accepted by the public.

‘Acceptability’ is a multifaceted concept influenced by various factors, reflecting how individuals perceive an intervention as appropriate, based on their cognitive and emotional responses either before the intervention’s implementation (prospective acceptability) or after experiencing it (retrospective acceptability).^17^ Unlike clinical interventions, which rely on individual consent, population-level interventions necessitate broad public approval before they can be implemented. Low public acceptance can lead to low political backing, reducing the likelihood of implementation, even when an intervention has been scientifically validated as effective.^18^ Thus, understanding public acceptability early in the planning process is crucial for informed and democratic decision-making.^19^^,^^20^

The NIL framework is increasingly employed in research to assess public acceptability, with emerging evidence revealing patterns that occasionally diverge from theoretical expectations. This review aims to synthesize available evidence, providing a comprehensive overview of how the NIL framework has been applied, identifying key trends, and offering insights into the factors influencing acceptability. For this scoping review, we define ‘acceptability’ as the extent to which interventions are perceived as agreeable by the target population (general public).

The primary research question guiding this review is: What is the current state of evidence regarding the application of the NIL framework in assessing public acceptability of diet and physical activity interventions within empirical research? The review will address the following subquestions:

1. What type of studies have utilized the NIL framework to assess public acceptability, and in what ways?
2. How is the concept of public acceptability of interventions defined and measured in studies using NIL framework?
3. Are there observable trends in acceptability outcomes based on the different levels of the NIL framework? What similarities and differences exist in acceptability across studies employing the NIL framework?
4. What factors are identified as influencing the public acceptability of interventions in studies using the NIL framework?

## Methods

This scoping review was conducted following the Joanna Briggs Institute (JBI) protocol for scoping reviews,^21^ which is based on Arksey and O’Malley’s methodological framework.^22^ For reporting, we adhered to the Preferred Reporting Items for Systematic Reviews and Meta-Analyses extension for Scoping Reviews (PRISMA-ScR) guidelines.^23^

### Identifying relevant studies

To identify relevant studies, we utilized the Population/Concept/Context (PCC) framework, as recommended by JBI, to guide the search strategy (see Table 1).^24^ A comprehensive search was conducted across multiple electronic databases, including PubMed, Scopus, Medline, Embase, Science Direct, and Web of Science. The initial search strategy was developed by the reviewer (S.R.) and then refined in consultation with an experienced librarian (Vicky Duncan), to ensure comprehensive literature coverage. To maximize sensitivity and avoid prematurely excluding relevant studies, we deliberately limited our search strategy to terms directly related to the NIL framework. This approach ensured the comprehensive capture of all studies that explicitly referenced or conceptually engaged with the NIL or its underlying principles. Additionally, relevant studies were identified through hand-searching key journals such as *the Journal of Ethics* and *Public Health Ethics*, reviewing the bibliographies of included articles and forward citation tracing to examine studies that cited the NIL. The initial search was conducted on 4 January 2024, and the most recent update was completed on 19 October 2024. Citations were managed using Zotero reference management software version 6.0.37,^25^ then exported to Covidence^26^ for automatic duplicate removal, screening, and data extraction.

**Table 1:** PCC framework for identifying the main concepts of the scoping review.

**PCC element**	**Definition**
**P**opulation	All individuals (no restriction)
**C**oncept	Acceptability of interventions targeting diet and physical activity
**C**ontext	Nuffield’s Intervention Ladder framework

### Study selection

Studies were eligible for inclusion if they explicitly applied the NIL framework to measure public acceptability of diet and physical activity–related interventions. During the title and abstract screening phase, we applied broad inclusion criteria, selecting all studies that employed the NIL framework to assess intervention acceptability for full-text review. Only peer-reviewed studies published in English from 2007 onwards (the year the NIL framework was introduced) were eligible for inclusion. During full-text screening, additional exclusion criteria were applied. Studies were excluded if they did not specifically assess acceptability, focused on interventions unrelated to diet or physical activity, or did not assess public acceptability. Studies focusing exclusively on intervention deliverers such as policy makers or other decision-makers were excluded. Eligible studies included empirical research such as quantitative, qualitative, and mixed-methods studies, as well as systematic and scoping reviews, to capture a comprehensive understanding of how the NIL has been applied across different study types. Opinion pieces, discussion articles, and studies that cited the NIL framework without substantive application were excluded. To maintain a consistent standard of evidence and focus on empirical applications of the NIL, we limited our inclusion to peer-reviewed publications, thereby excluding grey literature.

### Data charting and reporting

Data were charted using a customized template in Covidence.^26^ The form captured key study characteristics and relevant information aligned with the research questions, including target intervention, target population, methodological details, method of acceptability assessment, method of application of NIL framework, and key findings. The charting process was iterative, allowing the template to be refined and updated as new insights emerged throughout the review. Reviewer one (S.R.) independently screened studies and extracted data, consulting reviewer two (N.M.) only when uncertainties arose during the process.

Following the data charting phase, we synthesized the findings from the included studies according to distinct themes that we developed based on the objectives. In studies that involved multiple target populations (e.g. intervention receivers such as the general public and intervention deliverers such as policy influencers or other decision-makers), we focused specifically on the findings related to the acceptability among intervention receivers, the general public. The results were then organized thematically to address the specific subquestions outlined above.

## Results

### Search results

**Figure 2:**
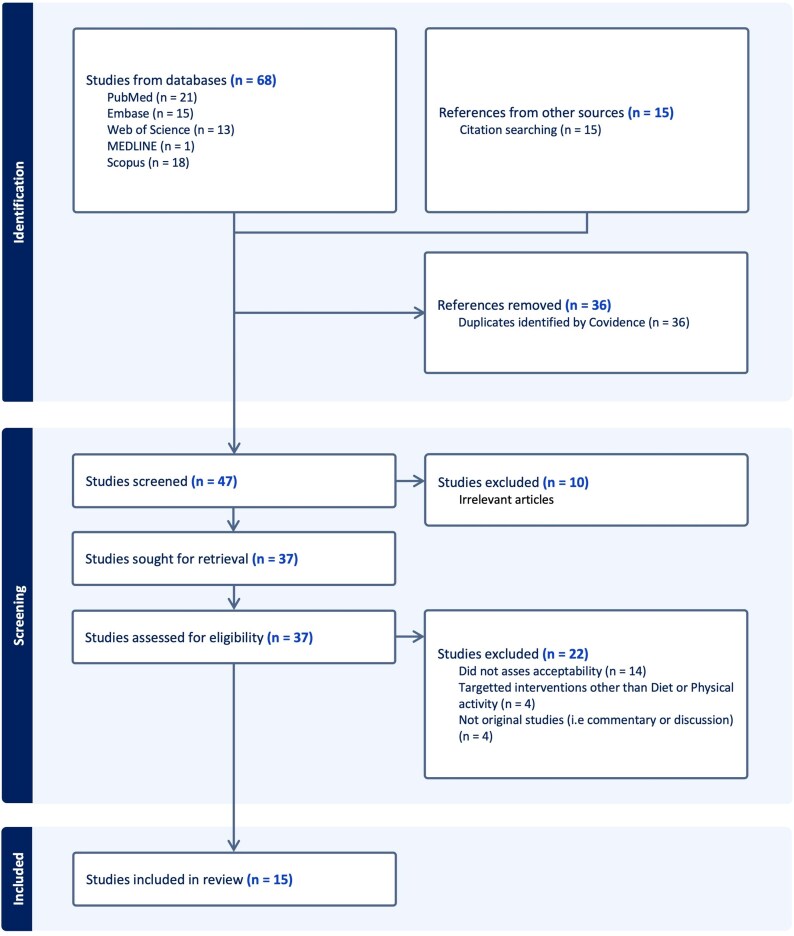
PRISMA (Preferred Reporting Items for Systematic Reviews and Meta-Analyses) flow chart of study selection procedure and results.

The search yielded 83 records. After removing duplicates, 47 studies proceeded to the title and abstract screening. During this, 10 studies were excluded as they were irrelevant to this scoping review (e.g. did not mention NIL). The remaining 37 studies underwent a full-text review, resulting in the exclusion of 22 studies. Reasons for exclusion included: not assessing acceptability (*n* = 14), focusing on interventions unrelated to diet and physical activity (*n* = 4), or being discussion/commentary articles (*n* = 4). At the end, 15 studies met the inclusion criteria and were included in the final detailed review^27–41^ (see Fig. 2).

### Overview of included studies

The review includes 15 studies published between 2013 and 2024, comprising 11 cross-sectional studies,^27–29^^,^^31^^,^^34–36^^,^^38–41^ two systematic reviews,^30^^,^^37^ one qualitative study,^32^ and one mixed-methods study (Table 2).^33^ The majority of the included studies were published within the public health domain (8/15),^27^^,^^28^^,^^30^^,^^31^^,^^33^^,^^35^^,^^37^^,^^38^ followed by three in Nutrition,^29^^,^^34^^,^^40^ two in Urban Planning and Transport,^39^^,^^41^ and one each in health promotion^32^ and Physical Activity.^36^ Geographically, eight studies were conducted in Canada,^27^^,^^28^^,^^31^^,^^35^^,^^36^^,^^39–41^ four in Europe,^29^^,^^32^^,^^34^^,^^38^ and one in Australia.^33^ The two systematic reviews were authored by researchers based in Europe and covered studies from a wide range of countries, including the United States, Canada, Australia, New Zealand, the United Kingdom, Central Asia, and other European and Asian countries.^30^^,^^37^

**Table 2:** Overview of studies included in the review.

Authors, year of publication	Study design	Country	Intervention type	Study population	Sample	Data collection method	Definition of Acceptability	Acceptability measurement	Use of NIL framework	Levels of NIL represented
*Diepeveen* et al*. (2013)*	Systematic review	United Kingdom	Diet and Physical activity	General public	200 studies	Systematic review	No formal definition or operationalization provided; acceptability was implied through terms like ‘support’ and “endorse	N/A	Interventions from the reviewed studies were grouped into three broad categories: providing information, guiding choice through incentives or disincentives, and restricting or eliminating choice	All levels except ‘do nothing or monitor the situation’
*Bos* et al. *(2015)*	Cross-sectional study	Netherland	Diet	General public (above 18 years)	1173 participants	Online survey	No formal definition or operationalization provided; acceptability was implied through terms like ‘acceptance’ and ‘agreement’	Measured using seven-point Likert-scale items ranging from ‘completely disagree’ to “completely agree	Interventions were grouped as providing information, guiding choice through (dis)incentives, and restricting choice.	Only ‘provide information’, ‘guide choice through disincentives’, and ‘restricting choice’
*Stok* et al. *(2016)*	Cross-sectional study	Poland, Portugal, the Netherlands, and the United Kingdom	Diet	General Public (adolescents)	2764 participants	In-person survey	No formal definition or operationalization provided; acceptability was implied through terms like ‘acceptance’ and ‘endorsement’	Measured using five-point Likert-scale items ranging from ‘strongly disagree’ to ‘strongly agree’	The study used the NIL to guide the selection of strategies with varying intrusiveness but did not explicitly apply any categorization labels.	All except ‘guide choice by changing default policy’ and ‘do nothing or monitor the situation’
*Haynes* et al. *(2017)*	Mixed-methods study	Australia	Diet and physical activity	Stakeholders including general public	158 documents	Content analysis	Stakeholder support was defined as the ‘frequency of recommendation’ for a policy option	Measured using the frequency with which policy options were recommended in stakeholder submissions	The study used the NIL and Griffiths and West’s Balanced Ladder to categorize policy recommendations by intrusiveness and autonomy. Enable choice was coded as autonomy-enhancing, providing information as autonomy-increasing, and guiding choice through changing the default policy was categorized as neutral. Guiding choice through incentives or disincentives was coded as autonomy-reducing, while restricting or eliminating choice was categorized as autonomy-diminishing. A new neutral category, ‘building capacity’, was added, and recommendations that didn’t fit either framework were grouped as ‘guiding principles’.	All levels except ‘do nothing or monitor the situation’
*Bhawra* et al*. (2018)*	Cross-sectional study	Canada	Diet	General public	2729 participants	Online survey	No formal definition or operationalization provided; acceptability was implied through term ‘support’	Measured using response options including ‘Support’, ‘Neutral’, ‘Oppose’, or ‘Don’t Know’	NIL framework used in discussion to interpret findings; no explicit labelling	All except ‘enable choice’, ‘guide choice by changing defaults’, and ‘do nothing or monitor the situation’
*Edache* et al. *(2018)*	Cross-sectional study	Canada	Diet	General public	903 participants	Online survey	No formal definition or operationalization provided; acceptability was implied through term ‘support’	Measured using four-point Likert-scale items ranging from ‘strongly Oppose’ to ‘strongly Support’	The study categorized interventions as less intrusive (enable/educate) and more intrusive (incentives/disincentives, eliminate choice)	All except ‘guide choice by changing defaults’, ‘restrict choice’, and ‘do nothing or monitor the situation’
*Bélanger-Gravel* et al. *(2019)*	Cross-sectional study	Canada	Diet	General public	1000 participants	Telephone survey	No formal definition or operationalization provided; acceptability was implied through term ‘support’	Measured using four-point Likert-scale items ranging from ‘strongly disagree’ to ‘strongly agree’	The study categorized interventions according to the NIL, ranging from doing nothing to restricting choice	All except ‘eliminate choice’
*McGetrick* et al. *(2019)*	Cross-sectional study	Canada	Physical activity	General public and policy influencers	2400 participants	Online survey	No formal definition or operationalization provided; acceptability was implied through term ‘support’	Measured using four-point Likert-scale items ranging from ‘strongly Oppose’ to ‘strongly Support’	The study categorized different policy options using the NIL, ranging from providing information to restricting choice, based on their level of intrusiveness	All levels except ‘do nothing or monitor the situation’ and ‘eliminate choice’
*Kongats* et al. *(2019)*	Cross-sectional study	Canada	Diet	General public and policy influencers	2400 participants	Online survey	No formal definition or operationalization provided; acceptability was implied through term ‘support’	Measured using four-point Likert-scale items ranging from ‘strongly Oppose’ to ‘strongly Support’	The study categorized different policy options using the NIL, ranging from providing information to restricting choice, based on their level of intrusiveness	All levels except ‘do nothing or monitor the situation’ and ‘eliminate choice’
*Graham* et al. *(2020)*	Qualitative study	United Kingdom	Diet	Customers and business owners	45 participants	Focus group	No formal definition or operationalization provided; acceptability was implied through terms like ‘support’ and ‘feasibility’	Acceptability inferred from participants’ attitudes and perceived feasibility of interventions.	The study employed NIL as a guiding framework to devise intervention options of varying intrusiveness for discussion during the focus groups	All levels except ‘do nothing or monitor the situation’ and ‘guide choice by changing defaults’
*Jürkenbeck* et al. *(2020)*	Cross-sectional study	Germany	Diet	General public	1035 participants	Online survey	No formal definition or operationalization provided; acceptability was implied through terms like ‘opinion’ and ‘agreement’	Measured using five-point Likert-scale items ranging from ‘do not agree at all’ to ‘Totally agree’	The authors grouped interventions into three broad categories based on the NIL: decision support (monitoring the situation, providing information, and enabling choice), decision guidance (guiding choice through changing defaults, incentives, and disincentives), and decision restriction (restricting and eliminating choice). However, they did not explicitly apply these levels to the interventions assessed.	The corresponding levels of the interventions assessed are not clearly defined.
*Scheidmeir et al. (2022)*	Systematic review	Germany	Diet and physical activity	General public and policy influencers	49 studies	Systematic review	Defined as the ‘perception among stakeholders that the implementation of a given policy is agreeable, palatable or satisfactory’	N/A	Interventions were divided into three groups based on NIL: highly intrusive policies (including guiding choice through disincentives and restricting or eliminating choice), moderately intrusive policies (such as guiding choice through incentives and changing default options), and low intrusiveness policies (including providing information and enabling choice).	All levels except ‘do nothing or monitor the situation’
*Lambert-De Francesch* et al. *(2024)*	Cross-sectional study	Canada	Diet	General public	27 162 participants	Online and telephone survey	No formal definition or operationalization provided; acceptability was implied through term ‘agreement’	Measured using four-point Likert-scale items ranging from ‘Completely disagree’ to ‘Completely agree’	The study examines policy interventions representing three levels of the NIL: changing the default choice, restricting choice, and eliminating choice.	Only ‘guide choice by changing defaults’, ‘restrict choice’, and ‘eliminate choice’
*Ramamoorthy* et al. *(2024)*	Cross-sectional study	Canada	Diet and physical activity	General public	2133 participants	Online and telephone survey	Operationalized as the ‘level of agreement to implement each built environment intervention in their residential neighbourhood’	Measured using four-point Likert-scale items ranging from ‘Completely disagree’ to ‘Completely agree’	The study categorized different policy options using the NIL, ranging from providing information to eliminating choice, based on their level of intrusiveness	All levels except ‘do nothing or monitor the situation’
*Hosford* et al. *(2024)*	Cross-sectional study	Canada	Physical activity	General public	27 162 participants	Online and telephone survey	Defined as the ‘level of agreement for a specific policy intervention’	Measured using four-point Likert-scale items ranging from ‘Completely disagree’ to ‘Completely agree’	The study examines interventions representing five levels of the NIL—enable choice, guide choice through changing defaults, guide choice through disincentive, restrict choice, and eliminate choice	All levels except ‘do nothing or monitor the situation’, ‘provide information’, and guide choice through incentive

In terms of intervention focus, most studies (*n* = 9) addressed diet-related interventions,^27–29^^,^^31^^,^^32^^,^^34^^,^^35^^,^^38^^,^^40^ two focussed exclusively on physical activity,^36^^,^^41^ and four covered both diet and physical activity interventions.^30^^,^^33^^,^^37^^,^^39^ Regarding the study population, the majority (*n* = 10) assessed public acceptability exclusively, while the remaining studies involved both the public and intervention deliverers, such as policy influencers and business owners.^32^^,^^33^^,^^35–37^ One study used the term ‘stakeholders’ but did not clearly define who they were.^33^ However, it was apparent that the general public was one of the key groups targeted.

The following findings are presented in the Supplementary Appendix 2: (i) the types of studies that applied the NIL framework and (ii) how these studies defined and measured acceptability.

### Are there any trends in acceptability based on different levels of the Nuffield’s Intervention Ladder framework?

Overall, interventions that were the focus of reviewed studies received significant support, with distinct patterns emerging based on the level of intrusiveness as outlined by the NIL framework. Acceptability of the first level of the NIL, ‘doing nothing or monitoring the situation’, was explicitly assessed only in one study, which found an equal proportion of individuals both agreeing and disagreeing with the intervention.^27^

#### Higher acceptability for less intrusive interventions

Across all studies, less intrusive interventions, such as information provision and choice enablement, consistently garnered higher levels of public support. These interventions were seen as empowering individuals by providing information and the freedom to make choices.^32^^,^^33^ Interventions that provided information and guided choices were particularly well-received as they were perceived as both personally and societally effective.^29^

#### Mixed acceptability for moderately intrusive interventions

Moderately intrusive interventions, such as changing defaults, guiding choice through incentives and disincentives, received varying levels of support across different studies.^33^^,^^36^^,^^39^^,^^40^ Some studies found that default changes, such as altering the usual side dish option in restaurants, garnered higher support compared to less intrusive interventions.^40^^,^^41^ However, an opposite trend was also observed, particularly in interventions related to physical activity, where changes to defaults, such as building separate bike lanes and limiting speed at 40 km/hr in the entire city, received lower support than more intrusive measures.^39^ Incentives to promote physical activity were generally less supported; however, incentives to promote healthy eating were strongly welcomed. For example, tax credits for people engaged in regular physical activity or priority traffic lights for busses received lower support.^36^^,^^39^ Disincentives, such as raising prices on junk food, taxing sugary drinks, and imposing road tolls, typically garnered less support.^32^^,^^33^^,^^35^^,^^37^^,^^39^ Food taxes on unhealthy products were accepted by a higher proportion of the respondents when paired with tax reductions on healthier options.^28^^,^^34^ Additionally, acceptance of food taxation increased among lower socioeconomic groups when accompanied by complementary initiatives, such as using tax revenue from unhealthy foods to subsidize fruits and vegetables.^37^

#### Lower acceptability for highly intrusive interventions

Highly intrusive interventions, such as restrictions and eliminations of choice, were generally met with lower levels of acceptance.^27–29^^,^^35^^,^^36^^,^^39^^,^^41^ Resistance was particularly pronounced among individuals who felt that limiting personal choices infringed upon their autonomy, especially when they believe that individuals did not have sole responsibility for health outcomes.^32^ However, this does not imply more intrusive interventions were universally rejected. The acceptability of these interventions was contingent on the type and its context.^28–32^^,^^38^

#### Conditional acceptability of highly intrusive interventions targeting children, youth, and industries

The acceptability of more intrusive interventions, such as restrictions and eliminations, varied significantly depending on the target group and the setting. Interventions targeting children and youth were more readily accepted, even if they were highly intrusive.^27^^,^^28^^,^^30^^,^^33^^,^^34^^,^^36^ These included mandatory physical activity in schools and preschools, bans on unhealthy food marketing targeting children, and nutrition standards for school cafeterias, all of which received strong public support.^28^^,^^34^^,^^36^ Similarly, more intrusive interventions directed at the food industries and businesses, such as regulations on food marketing and mandatory product reformulation, also received higher public support.^27^^,^^29^^,^^33^ These policies were often viewed as fair, contributing to their broader acceptance.^29^

### What key factors influence the acceptability of interventions in these studies?

#### Gender

Women consistently demonstrated greater acceptability for interventions, regardless of their type or level of intrusiveness.^27^^,^^30^^,^^37–41^ They also tended to agree with implementing interventions that they perceived as effective and appropriate.^37^

#### Age

Older individuals generally expressed greater support for interventions of all types.^28^^,^^30^^,^^37^ However, this trend was not consistent across all studies. One study showed a negative association with age, where younger individuals were more likely to support interventions promoting active travel.^41^ Another study reported no significant relationship between age and support for diet or physical activity-related interventions.^39^

#### Socioeconomic influences

Several studies focussed on specific socioeconomic indicators, such as income and education, rather than assessing socioeconomic status as a comprehensive measure. Higher levels of education and health literacy were consistently associated with greater acceptability of interventions.^28^^,^^37^^,^^39^^,^^40^ The influence of education on intervention acceptability appeared to be mediated by unhealthy eating habits, as individuals with higher educational qualifications were more likely to adopt healthier eating habits.^27^ This, in turn, positively influenced their acceptance of interventions promoting healthier behaviours. However, the relationship between income and intervention acceptability was less clear. Some studies found that lower-income groups were more supportive of interventions,^37^^,^^39–41^ whereas others reported no significant correlation between income and acceptability.^27^^,^^30^^,^^38^

#### Race/ethnicity/immigrant status

Support for interventions varied across racial and ethnic groups.^28^ Indigenous individuals and Immigrants tended to express higher levels of support, including for more intrusive interventions.^39–41^

#### Perceived intrusiveness, fairness, and effectiveness

Individuals who placed a high value on personal autonomy and believed that government intervention should not extend into personal choices were less likely to support, and more likely to oppose, such measures.^27^ However, interventions that enhanced personal autonomy—such as providing information or enabling choice—tended to be received more favourably.^33^ The perceived effectiveness of interventions, both in terms of personal benefits (such as improving physical fitness or managing weight) and broader societal impact (like reducing obesity rates), served as a key mediator in the link between intervention intrusiveness and acceptability. Less intrusive interventions were often perceived as more effective by individuals, leading to greater support for them.^29^^,^^32^ However, some interventions perceived as highly effective in achieving their intended goals were better received, regardless of their intrusiveness. Additionally, interventions perceived as fair, for example, those targeting food manufacturers, were more likely to gain support, as people are more inclined to back initiatives that align with values of equity and justice.^29^

#### Geography and social-political orientation

Geographic location influenced the acceptability of interventions, with cultural and political factors playing a significant role^28^^,^^30^^,^^38^^,^^40^^,^^41^ In Canada, differences in acceptability were observed across cities and provinces.^36^^,^^40^^,^^41^ Interestingly, studies exploring the impact of neighbourhood-level factors did not reveal any strong associations.^39^^,^^41^

#### Target behaviour

Intervention acceptability was often influenced by the behaviour targeted for change, with individuals not already engaged in the desired behaviours being less likely to support such interventions***.*** In most studies, individuals with less healthy dietary habits were less likely to support interventions aimed at promoting healthy eating.^27^^,^^30^^,^^38^ Similarly, individuals who commuted by car were less supportive of measures to encourage active travel compared to those who walked, cycled, or used public transport.^41^

Interventions aimed at promoting healthy behaviours were generally more acceptable, even when they involved higher levels of intrusiveness.^29^^,^^38^ People tended to view these interventions as effective, which increased their willingness to support them, compared to interventions focused on discouraging unhealthy behaviours.^29^ However, when targeting unhealthy behaviours, less intrusive measures were likely to gain acceptance.^38^

## Discussion

### Main findings of this study

This scoping review examined the use of the NIL framework in assessing public acceptability of diet and physical activity interventions. Fifteen studies published between 2013 and 2024 were included (NIL framework was first introduced in 2007), with most focussing on diet-related interventions. All studies assessed prospective acceptability—public support or opposition before intervention implementation, typically through online surveys using ordinal and Likert-scale questionaries. The evidence included in our review is primarily from high-income countries, which was not a deliberate exclusion but rather reflects the current extent of the application of the NIL framework and the available literature. This highlights that the NIL framework has gained particular popularity in Western contexts.

The application of the NIL framework in the reviewed studies primarily revolved around classifying interventions according to their level of intrusiveness. In many cases, the NIL framework was integrated early in the design phase, guiding intervention selection, or was applied post hoc to analyze the variation in acceptability as per their level of intrusiveness. Although the studies did not directly report the specific level of intrusiveness of interventions to participants, the NIL framework was pivotal in interpreting responses within the context of intrusiveness.

Some studies focussed on interventions at specific levels of the NIL, while others adapted the framework by merging levels. In one study, the NIL was used alongside alternative model, the ‘Balanced Intervention Ladder’ proposed by Griffiths and West (2015), which addresses limitations in the original model by recognizing that some interventions may not only restrict but also enhance autonomy.^42^ Another notable observation was the minimal inclusion of level 1, ‘doing nothing’ or ‘monitoring the situation’. This level was often omitted, as it was not considered an ‘actual’ intervention but rather the status quo. This exclusion raises conceptual questions. Although inaction and monitoring are often regarded as minimally intrusive strategies, they still involve deliberate choices and serve specific functions in public health.^43^ In certain situation, inaction may be morally indefensible, undermining fairness, justice, and equity.^44^ For example, neglecting to address the targeted marketing of unhealthy foods to children can be viewed as unethical, given that children cannot make fully informed choices. Similarly, failing to act during a pandemic could further infringe on individual freedom.

Trends in public acceptability largely align with the NIL framework, indicating that less intrusive interventions received higher support. These interventions, which empower individuals to make informed choices without restrictions, are considered effective and respectful of autonomy. However, interventions involving changes in defaults, incentives, and disincentives often elicited mixed reactions, as they were sometimes seen as manipulative, akin to ‘nudging’. Incentives were generally less accepted, as they can be perceived as coercive, particularly when they push individuals towards behaviours they might not otherwise choose, thus undermining their sense of autonomy. Although intended to offer equal opportunities, incentives may disproportionately affect those with financial disadvantages. For these individuals, the pressure to accept incentives may feel like a compromise on their freedom of choice.^44–47^ In contrast, disincentives were sometimes more accepted when paired with complementary initiatives that offered additional benefits. As interventions became more intrusive, such as restrictions or mandates, public support generally declined. Yet, restrictive policies targeting children and youth or food industries were often viewed more favourably, due to the perceived collective benefit and protection of vulnerable populations.^44^

Gender, age, education, and cultural identity significantly influenced public support, with women, older individuals, those with higher educational qualification, and individuals with stronger health literacy generally showing higher levels of support. Unlike education, the relationship between income and intervention acceptability was equivocal, making it challenging to draw definitive conclusions. Self-identified Indigenous individuals and immigrants expressed greater support across all levels of intervention. Acceptability also varied across and within countries, shaped by cultural and sociopolitical factors. Personal behaviours influenced support, with people often opposing interventions that could disrupt their established routines or convenience, demonstrating tendencies consistent with NIMBYism. Interestingly, people preferred interventions promoting healthy behaviours over those that discouraged unhealthy ones. Support for interventions targeting unhealthy behaviours increased when they were less intrusive.

### What is already known

To our knowledge, no existing reviews have specifically evaluated the application of the NIL framework in empirical studies assessing the acceptability of health interventions. The NIL framework theorizes a relationship between the intrusiveness of interventions and public acceptability, emphasizing the role of personal autonomy and freedom. While several individual studies have employed this framework to interpret public responses based on intervention intrusiveness, a comprehensive synthesis of how the NIL framework has been applied across the literature is lacking. The primary objective of this review was to examine how the NIL framework has been applied in assessing the acceptability of interventions aimed at diet and physical activity, rather than to synthesize findings on acceptability outcomes more broadly.

### What this study adds

By synthesizing findings from 15 studies published between 2013 and 2024, this scoping review provides novel insights into the current application of the NIL framework and assesses whether empirical findings align with its theoretical hypotheses. Our review largely confirms the expected inverse relationship between intervention intrusiveness and public acceptability. Importantly, however, it also highlights that this relationship is more nuanced than the framework suggests. Public acceptability is influenced by a variety of contextual and demographic factors not explicitly accounted for by the NIL framework, indicating areas where the framework could be further developed to better capture the complexity of public attitudes towards health interventions.

### Limitations of this study

This scoping review is the first to comprehensively explore the application of the NIL framework in assessing the public acceptability of diet and physical activity interventions. As a scoping review, this study did not include a formal quality assessment of the included studies, nor did we perform a quantitative synthesis. The evidence presented remains qualitative and descriptive, aiming to provide broad insights into the research landscape rather than definitive or statistically robust conclusions. The included studies vary in design, methodology, and sample size, which may affect the generalizability and strength of the overall findings. Finally, the review is limited by its focus on only two domains, diet and physical activity, which excludes potentially relevant applications of the NIL in other public health areas. The lack of similar reviews for comparison also restricts the ability to situate our findings within a broader evidence base.

## Conclusion

This review highlights the application of the NIL framework in assessing public acceptability of diet and physical activity interventions. The framework has been most commonly used in cross-sectional survey studies published in the public health and nutrition domains, with growing adoption in Western, high-income settings. Acceptability was typically measured prospectively, using online surveys and Likert-scale instruments. Trends across studies indicate that less intrusive interventions such as those that provide information or enable choice are generally more acceptable to the public. While more intrusive interventions received less support overall, they were more acceptable when targeting vulnerable populations (such as children) or industries and when perceived as fair or effective. These findings suggest that while the NIL framework remains a useful tool for categorizing and interpreting public responses to interventions, greater attention to contextual and demographic factors is needed to fully understand what drives acceptability.

## Supplementary Material

revised_Supplementary_Appendix_2_fdaf156

## Data Availability

The data underlying this article are available in the article. Any additional data that support the study’s findings are available from the corresponding author upon request.

## Notes

**Sahana Ramamoorthy**, PhD Candidate and Research Technician

**Nazeem Muhajarine**, Professor & Director

**Lise Gauvin**, Professor & Associate Scientific Director

## References

[1] Wright B, Bragge P. Interventions to promote healthy eating choices when dining out: A systematic review of reviews. Br J Health Psychol 2018;23:278–95. 10.1111/bjhp.12285 29178363

[2] Vos M, Deforche B, Van Kerckhove A et al. Intervention strategies to promote healthy and sustainable food choices among parents with lower and higher socioeconomic status. BMC Public Health 2022;22:2378. 10.1186/s12889-022-14817-y 36536355 PMC9761028

[3] Hendry VL, Almiron-Roig E, Monsivais P et al. Interventions to promote healthy eating: A systematic scoping review of regulatory approaches. Lancet 2013;382:S45. 10.1016/S0140-6736(13)62470-8

[4] Bucher T, Collins C, Rollo ME et al. Nudging consumers towards healthier choices: A systematic review of positional influences on food choice. Br J Nutr 2016;115:2252–63. 10.1017/S0007114516001653 27185414

[5] Carter SM, Rychetnik L, Lloyd B et al. Evidence, ethics, and values: A framework for health promotion. Am J Public Health 2011;101:465–72. 10.2105/AJPH.2010.195545 21233436 PMC3036693

[6] Dixon BN, Ugwoaba UA, Brockmann AN et al. Associations between the built environment and dietary intake, physical activity, and obesity: A scoping review of reviews. Obes Rev 2021;22:e13171. 10.1111/obr.13171 33369097 PMC8629168

[7] de Vet E, de Ridder DTD, de Wit JBF. Environmental correlates of physical activity and dietary behaviours among young people: A systematic review of reviews. Obes Rev 2011;12:e130–42. 10.1111/j.1467-789X.2010.00784.x 20630024

[8] Cappuccio FP, Capewell S, Lincoln P et al. Policy options to reduce population salt intake. BMJ 2011;343:d4995. 10.1136/bmj.d4995 21835876

[9] Gasana J, O’Keeffe T, Withers TM, Greaves CJ. A systematic review and meta-analysis of the long-term effects of physical activity interventions on objectively measured outcomes. BMC Public Health 2023 23(1):1697, 10.1186/s12889-023-16541-7. 37660119 PMC10474717

[10] Greaves CJ, Sheppard KE, Abraham C et al. Systematic review of reviews of intervention components associated with increased effectiveness in dietary and physical activity interventions. BMC Public Health 2011;11:119. 10.1186/1471-2458-11-119 21333011 PMC3048531

[11] Summerbell CD, Waters E, Edmunds LD et al. Interventions for preventing obesity in children. Cochrane Database Syst Rev 2005:CD001871. 10.1002/14651858.CD001871.pub2 16034868

[12] Jepson RG, Harris FM, Platt S et al. The effectiveness of interventions to change six health behaviours: A review of reviews. BMC Public Health 2010;10–538. 10.1186/1471-2458-10-538 PMC2944371 20825660

[13] Hurlimann T, Peña-Rosas JP, Saxena A et al. Ethical issues in the development and implementation of nutrition-related public health policies and interventions: A scoping review. PLoS One 2017;12:e0186897. 10.1371/journal.pone.0186897 29073186 PMC5658098

[14] Nuffield Council on Bioethics . *Public Health: Ethical Issues*. London: Cambridge Publishers, Ltd, 2007, 191.

[15] Smith MJ, Gauthier K. The intervention ladder and the ethical appraisal of systemic public health interventions. J Med Ethics 2024;50:698–9. 10.1136/jme-2024-110161 38969485

[16] Whittall H . A closer look at the Nuffield Council on Bioethics - Hugh Whittall, 2008. Clin Ethics 2008;3:199–204. 10.1258/ce.2008.008034

[17] Sekhon M, Cartwright M, Francis JJ. Acceptability of healthcare interventions: An overview of reviews and development of a theoretical framework. BMC Health Serv Res 2017;17:88. 10.1186/s12913-017-2031-8 28126032 PMC5267473

[18] Lee K, van Nassau F, Grunseit A et al. Scaling up population health interventions from decision to sustainability – A window of opportunity? A qualitative view from policy-makers. Health Res Policy Syst 2020;18:118. 10.1186/s12961-020-00636-3 33036633 PMC7547476

[19] Page BI, Shapiro RY. Effects of public opinion on policy. Am Polit Sci Rev 1983;77:175–90. 10.2307/1956018

[20] Shapiro RY . Public opinion and American democracy. Public Opin Q 2011;75:982–1017. 10.1093/poq/nfr053

[21] Peters M, Godfrey C, Mcinerney P et al. The Joanna Briggs institute reviewers’ manual 2015: Methodology for JBI scoping reviews. In The Joanna Briggs Institute 2015:1–24.

[22] Arksey H, O’Malley L. Scoping studies: Towards a methodological framework. Int J Soc Res Methodol 2005;8:19–32. 10.1080/1364557032000119616

[23] Tricco AC, Lillie E, Zarin W et al. PRISMA extension for scoping reviews (PRISMA-ScR): Checklist and explanation. Ann Intern Med 2018;169:467–73. 10.7326/M18-0850 30178033

[24] Pollock D, Peters MDJ, Khalil H et al. Recommendations for the extraction, analysis, and presentation of results in scoping reviews. JBI Evid Synth 2023;21:520–32. 10.11124/JBIES-22-00123 36081365

[25] Zotero [computer program]. Version 6.0. Fairfax, VA, USA: Corporation for Digital Scholarship. Available from: https://www.zotero.org

[26] Covidence systematic review software. Veritas Health Innovation, Melbourne, Australia. https://www.covidence.org

[27] Bélanger-Gravel A, Desroches S, Janezic I et al. Pattern and correlates of public support for public health interventions to reduce the consumption of sugar-sweetened beverages. Public Health Nutr 2019;22:3270–80. 10.1017/S1368980019002076 31544722 PMC10260561

[28] Bhawra J, Reid JL, White CM et al. Are young Canadians supportive of proposed nutrition policies and regulations? An overview of policy support and the impact of socio-demographic factors on public opinion. Can J Public Health Rev Can Sante Publique 2018;109:498–505. 10.17269/s41997-018-0066-1 PMC6964476 29981092

[29] Bos C, Lans IVD, Van Rijnsoever F et al. Consumer acceptance of population-level intervention strategies for healthy food choices: The role of perceived effectiveness and perceived fairness. Nutrients 2015;7:7842–62. 10.3390/nu7095370 26389949 PMC4586565

[30] Diepeveen S, Ling T, Suhrcke M et al. Public acceptability of government intervention to change health-related behaviours: A systematic review and narrative synthesis. BMC Public Health 2013;13:756. 10.1186/1471-2458-13-756 23947336 PMC3765153

[31] Edache IY, Kakinami L, Alberga AS. Weight bias and support of public health policies. Can J Public Health 2021;112:758–65. 10.17269/s41997-020-00471-7 33990876 PMC8225739

[32] Graham F, Barker M, Menon M et al. Acceptability and feasibility of a café-based sustainable food intervention in the UK. Health Promot Int 2020;35:1507–18. 10.1093/heapro/daaa027 32243498

[33] Haynes E, Hughes R, Reidlinger DP. Obesity prevention advocacy in Australia: An analysis of policy impact on autonomy. Aust N Z J Public Health 2017;41:299–305. 10.1111/1753-6405.12660 28371184

[34] Jürkenbeck K, Zühlsdorf A, Spiller A. Nutrition policy and individual struggle to eat healthily: The question of public support. Nutrients 2020;12:516. 10.3390/nu12020516 32085503 PMC7071418

[35] Kongats K, McGetrick JA, Raine KD et al. Assessing general public and policy influencer support for healthy public policies to promote healthy eating at the population level in two Canadian provinces. Public Health Nutr 2019;22:1492–502. 10.1017/S1368980018004068 30782230 PMC10260847

[36] McGetrick JA, Kongats K, Raine KD et al. Healthy public policy options to promote physical activity for chronic disease prevention: Understanding Canadian policy influencer and general public preferences. J Phys Act Health 2019;16:565–74. 10.1123/jpah.2018-0020 31170864

[37] Scheidmeir M, Kubiak T, Luszczynska A et al. Acceptability of policies targeting dietary behaviours and physical activity: A systematic review of tools and outcomes. Eur J Pub Health 2022;32:iv32–49. 10.1093/eurpub/ckac053 36444105 PMC9897019

[38] Stok FM, de Ridder DTD, de Vet E et al. Hungry for an intervention? Adolescents’ ratings of acceptability of eating-related intervention strategies. BMC Public Health 2016;16:5. 10.1186/s12889-015-2665-6 26729328 PMC4700578

[39] Ramamoorthy S, Gauvin L, Muhajarine N. Acceptability of built environment interventions to improve healthy eating and physical activity among city dwellers in Saskatchewan, Canada: THEPA findings from a local context. Cities Health 2024;8:345–59. 10.1080/23748834.2024.2304926

[40] Lambert-De Francesch J, Saint-Onge K, Muhajarine N et al. Sociodemographic characteristics help predict Canadian urbanites’ acceptability of restaurant food environment policies. Front Nutr 2024;11:1360360. 10.3389/fnut.2024.1360360 38746940 PMC11091330

[41] Hosford K, Winters M, Saint-Onge K et al. Acceptability of built environment interventions to support active travel in 17 Canadian metropolitan areas: Findings from the THEPA study. Sustain Transp Livability 2024;1:2314024. 10.1080/29941849.2024.2314024

[42] Griffiths PE, West C. A balanced intervention ladder: Promoting autonomy through public health action. Public Health 2015;129:1092–8. 10.1016/j.puhe.2015.08.007 26330372

[43] Dawson AJ . Snakes and ladders: State interventions and the place of liberty in public health policy. J Med Ethics 2016;42:510–3. 10.1136/medethics-2016-103502 27215764

[44] Byskov MF . Qualitative and quantitative interpretations of the least restrictive means. Bioethics 2019;33:511–21. 10.1111/bioe.12548 30657601 PMC6590346

[45] Lemken D, Wahnschafft S, Eggers C. Public acceptance of default nudges to promote healthy and sustainable food choices. BMC Public Health 2023;23:2311. 10.1186/s12889-023-17127-z 37993839 PMC10664270

[46] Giubilini A., Giubilini A. Vaccination policies and the principle of least restrictive alternative: an intervention ladder. In: Giubilini A, editor. The Ethics of Vaccination [Internet]. Cham: Springer International Publishing; 2019 [cited 2024 Oct 9]. p. 59–93. 10.1007/978-3-030-02068-2_3

[47] Krubiner CB, Merritt MW. Which strings attached: Ethical considerations for selecting appropriate conditionalities in conditional cash transfer programmes. J Med Ethics 2017;43:167–76. 10.1136/medethics-2016-103386 27707877

