# Supplementary material for: A scoping review of studies applying the Nuffield’s ‘intervention ladder’ framework to assess the acceptability of diet and physical activity interventions
Source: J Public Health (Oxf). 2025 Dec 11;48(1):172–84. doi: 10.1093/pubmed/fdaf156 (PMC13016987; doi:10.1093/pubmed/fdaf156)
Supplement: revised_Supplementary_Appendix_2_fdaf156 [file revised_supplementary_appendix_2_fdaf156.docx]

**A scoping review of studies applying the Nuffield’s “Intervention Ladder” framework to assess the acceptability of diet and physical activity interventions**

***Supplementary Appendix 2***

**What type of studies have applied the NIL framework to assess acceptability, and in what ways**?

Our review revealed a diverse application of the NIL framework across different study designs, despite the small sample size. The most common design was the cross-sectional survey, used in 11 out of 15 studies. Of these, four studies collected data exclusively through online surveys,^1–4^ one utilized in-person data collection,^5^ another relied solely on telephone survey,^6^ and five employed a combination of online and telephone methods.^7–11^

All cross-sectional studies assessed acceptability prospectively, focusing on acceptability of interventions prior to their implementation. In these survey-based studies, the NIL framework was generally applied from the study design phase onward, guiding the selection of interventions for inclusion in survey questionnaires. During the analysis phase, interventions were coded according to their level of intrusiveness for further examination. However, not all studies comprehensively covered all levels of the NIL. Many studies focussed on intervention acceptability within just one or a few levels of the NIL, while others grouped the levels or adapted the framework with few modifications. For example, Jürkenbeck *et al.* (2020) divided interventions into three broad categories, decision support, guidance, and restrictions, and used adjusted labels, such as “simplified choice” instead of “enable choice” and “nudge” instead of “changing default”.^4^ One study included both diet and physical-activity interventions representing seven levels of the NIL, excluding the first level-doing nothing or monitoring the situation,^9^ while another study covered diet-related interventions representing the complete continuum of the NIL.^6^ Bhawra *et al.* (2018) utilized the NIL framework exclusively in the discussion, using it to interpret and contextualize their findings in relation to the intrusiveness of interventions.^1^

In the qualitative study by Graham *et al.* (2020), the NIL framework was used to structure focus group discussions that explored participants’ views on point-of-choice interventions promoting healthy and sustainable food options within a university setting.^12^ The focus groups proposed intervention options across seven levels of the NIL, excluding ‘doing nothing or monitoring the situation’. In Haynes *at al.* (2017) mixed-methods approach, the NIL framework was integrated with the “balanced intervention ladder”^13^ to analyze government policy submissions.^14^ Policies were categorized based on their ‘nature’ of impact on individual autonomy, such as autonomy-neutral, autonomy-reducing, autonomy-diminishing, autonomy-increasing and autonomy-enhancing. Both studies assessed acceptability prospectively.

In the two systematic reviews, the NIL framework was applied at the analysis stage to synthesize findings from multiple studies. Interventions from different studies were categorized according to their level of intrusiveness, allowing the authors to identify patterns and trends in how varying levels of intrusiveness influenced public support. Diepeveen *at al.* (2013) classified identified interventions into one of the five levels of the NIL, disregarding the remaining levels, i.e. enabling choice or guide choice by changing default policies.^15^ Scheidmeir et al. (2022) classified policies as per the NIL and grouped them into highly intrusive policies (guiding choice through disincentives and eliminating/ restricting choice), moderately intrusive policies (guiding choice through incentives and by changing the default policy) and lower intrusive policies (providing information and enabling choice).^16^

**How is the concept of acceptability defined and measured in these studies?**

Most studies did not explicitly define or operationalize the concept of acceptability. Instead, they used terms such as “support”, “agreement”, “opinion”, “favourability”, and “views” to convey the idea of acceptability. Two studies, however, operationalized acceptability as “the level of agreement to implement interventions”.^9,11^ In the cross-sectional surveys, most studies used 4-point ordinal scales or 5- or 7-point Likert scales to gauge participants’ responses, typically asking them to rate their “support” or “agreement” with various interventions. For example, McGetrick *et al.* (2019) used a 4-point ordinal scale ranging from “strongly oppose” to “strongly support”, aggregating responses to derive a net favourable score, categorizing interventions on a scale from ‘extremely favourable’ to ‘extremely unfavourable’.^8^

In the mixed-methods study, stakeholder support was measured by the ‘frequency of submitted recommendation’, assessing acceptability based on how often certain interventions were recommended by public.^14^ In the qualitive study, focus groups were used to explore acceptability and factors influencing food decisions.^12^ Acceptability was assesed by examining participants’ preferences, satisfaction with, willingness to pay, and perceived value—related to food. In the systematic review by Scheidmeir et al. (2022), they explicitly defined acceptability as “the perception among stakeholders that the implementation of a given policy is agreeable, palatable or satisfactory”, and examined how the studies in their review operationalized it. They found that only a limited number of studies offered a clear operationalization of acceptability and recommended that future research adopt more precise definitions based on existing acceptability frameworks.^16^

**Search strategy**

The following search strategy was developed for PubMed and adapted as needed for other databases to match database specific syntax.

("Nuffield Intervention Ladder"[Title/Abstract] OR "Intervention Ladder"[Title/Abstract] OR "Nuffield Council on Bioethics"[Title/Abstract] OR Nuffield*[Title/Abstract])

Limiters: English, from 2007 - 2024

References:

1. Bhawra J, Reid JL, White CM, Vanderlee L, Raine K, Hammond D. Are young Canadians supportive of proposed nutrition policies and regulations? An overview of policy support and the impact of socio-demographic factors on public opinion. Can J Public Health. 2018 Aug;109(4):498–505.

2. Bos C, Lans IVD, Van Rijnsoever F, Van Trijp H. Consumer Acceptance of Population-Level Intervention Strategies for Healthy Food Choices: The Role of Perceived Effectiveness and Perceived Fairness. Nutrients. 2015 Sep 15;7(9):7842–62.

3. Edache IY, Kakinami L, Alberga AS. Weight bias and support of public health policies. Can J Public Health. 2021 Aug 1; 112(4):758–65.

4. Jürkenbeck K, Zühlsdorf A, Spiller A. Nutrition Policy and Individual Struggle to Eat Healthily: The Question of Public Support. Nutrients. 2020 Feb 18;12(2):516.

5. Stok FM, de Ridder DTD, de Vet E, Nureeva L, Luszczynska A, Wardle J, et al. Hungry for an intervention? Adolescents’ ratings of acceptability of eating-related intervention strategies. BMC Public Health. 2016 Jan 5;16(1):5.

6. Bélanger-Gravel A, Desroches S, Janezic I, Paquette MC, Wals PD. Pattern and correlates of public support for public health interventions to reduce the consumption of sugar-sweetened beverages. Public Health Nutrition. 2019 Dec;22(17):3270–80.

7. Kongats K, McGetrick JA, Raine KD, Voyer C, Nykiforuk CI. Assessing general public and policy influencer support for healthy public policies to promote healthy eating at the population level in two Canadian provinces. Public Health Nutr. 2019 Jun;22(8):1492–502.

8. McGetrick JA, Kongats K, Raine KD, Voyer C, Nykiforuk CIJ. Healthy Public Policy Options to Promote Physical Activity for Chronic Disease Prevention: Understanding Canadian Policy Influencer and General Public Preferences. J Phys Act Health. 2019 Jun 27;16(7):565–74.

9. Ramamoorthy S, Gauvin L, Muhajarine N. Acceptability of built environment interventions to improve healthy eating and physical activity among city dwellers in Saskatchewan, Canada: THEPA findings from a local context. Cities & Health. 2024 May 3;8(3):345–59.

10. Lambert-De Francesch J, Saint-Onge K, Muhajarine N, Gauvin L. Sociodemographic characteristics help predict Canadian urbanites’ acceptability of restaurant food environment policies. Front Nutr. 2024;11:1360360.

11. Hosford K, Winters M, Saint-Onge K, Muhajarine N, Gauvin L. Acceptability of built environment interventions to support active travel in 17 Canadian metropolitan areas: findings from the THEPA study. Sustainable Transport and Livability. 2024 Dec 31;1(1):2314024.

12. Graham F, Barker M, Menon M, Holdsworth M. Acceptability and feasibility of a café-based sustainable food intervention in the UK. Health Promot Int. 2020 Dec 1;35(6):1507–18.

13. Griffiths PE, West C. A balanced intervention ladder: promoting autonomy through public health action. Public Health. 2015 Aug;129(8):1092–8.

14. Haynes E, Hughes R, Reidlinger DP. Obesity prevention advocacy in Australia: an analysis of policy impact on autonomy. Australian and New Zealand Journal of Public Health. 2017 Jun 1;41(3):299–305.

15. Diepeveen S, Ling T, Suhrcke M, Roland M, Marteau TM. Public acceptability of government intervention to change health-related behaviours: a systematic review and narrative synthesis. BMC Public Health. 2013 Aug 15;13:756.

16. Scheidmeir M, Kubiak T, Luszczynska A, Wendt J, Scheller DA, Meshkovska B, et al. Acceptability of policies targeting dietary behaviours and physical activity: a systematic review of tools and outcomes. European Journal of Public Health. 2022 Dec 1;32(Supplement_4):iv32–49.
